# Internal and External Validation of a Machine Learning Risk Score for Acute Kidney Injury

**DOI:** 10.1001/jamanetworkopen.2020.12892

**Published:** 2020-08-11

**Authors:** Matthew M. Churpek, Kyle A. Carey, Dana P. Edelson, Tripti Singh, Brad C. Astor, Emily R. Gilbert, Christopher Winslow, Nirav Shah, Majid Afshar, Jay L. Koyner

**Affiliations:** 1 Department of Medicine, University of Wisconsin, Madison; 2 Department of Medicine, The University of Chicago, Illinois; 3 Department of Population Health Sciences, University of Wisconsin, Madison; 4 Department of Medicine, Loyola University Medical Center, Maywood, Illinois; 5 Department of Medicine, NorthShore University Healthcare, Evanston, Illinois

## Abstract

**Importance:**

Acute kidney injury (AKI) is associated with increased morbidity and mortality in hospitalized patients. Current methods to identify patients at high risk of AKI are limited, and few prediction models have been externally validated.

**Objective:**

To internally and externally validate a machine learning risk score to detect AKI in hospitalized patients.

**Design, Setting, and Participants:**

This diagnostic study included 495 971 adult hospital admissions at the University of Chicago (UC) from 2008 to 2016 (n = 48 463), at Loyola University Medical Center (LUMC) from 2007 to 2017 (n = 200 613), and at NorthShore University Health System (NUS) from 2006 to 2016 (n = 246 895) with serum creatinine (SCr) measurements. Patients with an SCr concentration at admission greater than 3.0 mg/dL, with a prior diagnostic code for chronic kidney disease stage 4 or higher, or who received kidney replacement therapy within 48 hours of admission were excluded. A simplified version of a previously published gradient boosted machine AKI prediction algorithm was used; it was validated internally among patients at UC and externally among patients at NUS and LUMC.

**Main Outcomes and Measures:**

Prediction of Kidney Disease Improving Global Outcomes SCr-defined stage 2 AKI within a 48-hour interval was the primary outcome. Discrimination was assessed by the area under the receiver operating characteristic curve (AUC).

**Results:**

The study included 495 971 adult admissions (mean [SD] age, 63 [18] years; 87 689 [17.7%] African American; and 266 866 [53.8%] women) across 3 health systems. The development of stage 2 or higher AKI occurred in 15 664 of 48 463 patients (3.4%) in the UC cohort, 5711 of 200 613 (2.8%) in the LUMC cohort, and 3499 of 246 895 (1.4%) in the NUS cohort. In the UC cohort, 332 patients (0.7%) required kidney replacement therapy compared with 672 patients (0.3%) in the LUMC cohort and 440 patients (0.2%) in the NUS cohort. The AUCs for predicting at least stage 2 AKI in the next 48 hours were 0.86 (95% CI, 0.86-0.86) in the UC cohort, 0.85 (95% CI, 0.84-0.85) in the LUMC cohort, and 0.86 (95% CI, 0.86-0.86) in the NUS cohort. The AUCs for receipt of kidney replacement therapy within 48 hours were 0.96 (95% CI, 0.96-0.96) in the UC cohort, 0.95 (95% CI, 0.94-0.95) in the LUMC cohort, and 0.95 (95% CI, 0.94-0.95) in the NUS cohort. In time-to-event analysis, a probability cutoff of at least 0.057 predicted the onset of stage 2 AKI a median (IQR) of 27 (6.5-93) hours before the eventual doubling in SCr concentrations in the UC cohort, 34.5 (19-85) hours in the NUS cohort, and 39 (19-108) hours in the LUMC cohort.

**Conclusions and Relevance:**

In this study, the machine learning algorithm demonstrated excellent discrimination in both internal and external validation, supporting its generalizability and potential as a clinical decision support tool to improve AKI detection and outcomes.

## Key Points

**Question:**

What is the accuracy of a single-center machine learning algorithm for predicting acute kidney injury (AKI) when internally and externally tested?

**Findings:**

In this multicenter diagnostic study of approximately 500 000 admissions from 6 hospitals in 3 health systems, the machine learning algorithm had similarly high discrimination in both internal and external validation cohorts. Alert thresholds fired nearly a day and a half before the event.

**Meaning:**

These findings demonstrate that the AKI algorithm is generalizable to patients in the center in which it was derived and to patients from other hospitals, suggesting that implementation could prompt early identification and therapy aimed at decreasing preventable AKI.

## Teaser

This diagnostic study internally and externally validates a machine learning risk score for detecting acute kidney injury in hospitalized patients.

## Introduction

Acute kidney injury (AKI) is a common clinical syndrome in hospitalized patients and is associated with increased morbidity, mortality, and cost of care.^1,2,3^ Consensus criteria define AKI by either an increase in serum creatinine (SCr) concentration or a decrease in urine output.^4^ Biomarkers that detect AKI prior to these changes have been investigated for several years. However, to date, there has been limited large-scale validation and implementation of these tools. Detection of AKI prior to the changes in SCr concentration may provide a crucial window of opportunity to prevent further injury and allow clinicians to intervene in the hopes of improving patient outcomes.

While work on urinary and serum biomarkers of early AKI continues,^5,6,7^ several groups have reported on the accuracy of electronic health record–based risk scores that can identify AKI before changes in SCr concentration.^8,9,10,11,12,13,14^ The scope of these published algorithms has varied, with some focusing on only ward or intensive care unit (ICU) patients and others on postoperative AKI.^8,9,10,11,12,13,14^ Additionally, these algorithms range from rule-based, more parsimonious scores to complex, machine learning–based scores.^8,10,13^ However, regardless of the individual score, there has been limited external validation of these risk assessment tools. Our group has previously published a gradient boosted machine learning AKI prediction model for all hospitalized patients (ie, patients in the emergency department, ward, and ICU) using single-center data at the University of Chicago (UC).^10^ We subsequently simplified this risk score and clinically implemented the streamlined version to prompt early nephrology consultation as part of a single-center randomized controlled trial.^15^ In this study, we aim to both internally (at UC) and externally validate the simplified version of our AKI score using retrospective cohorts from independent health systems (Loyola University Medical Center [LUMC], Maywood, Illinois, and Northshore University Health System [NUS], Evanston, Illinois).

## Methods

### Study Population

We included 3 distinct adult (≥18 years) patient cohorts in this retrospective cohort study of prospectively collected data. All admitted adult patients at UC (an urban tertiary referral hospital) who were part of the validation cohort (2008 to 2016) in our previously published AKI algorithm development study^10^ were used for internal validation of the model. External validation was performed in adult patients admitted to LUMC (a suburban tertiary referral hospital) from 2007 to 2017 and NUS (a suburban 4-hospital health care network) from 2006 to 2016. Patients were excluded if they had no documented SCr concentration during their admission; had an initial admitting SCr concentration of at least 3.0 mg/dL (to convert to micromoles per liter, multiply by 88.4); had diagnosis codes for stage 4 or higher chronic kidney disease from any prior inpatient or outpatient encounter; developed Kidney Disease Improving Global Outcomes (KDIGO) stage 2 AKI (ie, SCr concentrations doubled) in a location other than the ward, emergency department, or ICU; or required kidney replacement therapy (KRT) within 48 hours of their first documented SCr measurement.^10^ The study protocol was approved by the UC, LUMC, and NUS institutional review boards with a waiver of informed consent based on minimal harm and impracticability. We followed the Transparent Reporting of a Multivariable Prediction Model for Individual Prognosis or Diagnosis (TRIPOD) reporting guideline.^16^

### Data Collection

Demographic characteristics, patient location data (eg, ward, emergency department, ICU, operating room), vital signs, laboratory values, and nurse documentation were accessed through the Clinical Research Data Warehouse at UC. A similar process was completed at LUMC and NUS through their respective data warehouses, and data from the external sites were transferred to UC for analysis under a data use agreement.

### AKI Definitions

We defined AKI by the SCr-based criteria from the KDIGO consensus definition.^4^ Baseline SCr concentration was defined as the admission SCr value and was updated on a rolling basis for 48-hour and 7-day criteria, as per the KDIGO guidelines.^4,9,10^

### Statistical Analysis

Patient characteristics, laboratory values, and outcomes were compared among the 3 cohorts (NUS, LUMC, and UC). These same factors were compared within the individual cohorts between patients who developed AKI and those who did not. We used *t* tests, Wilcoxon rank sum tests, analyses of variance, Kruskal-Wallis tests, and χ^2^ tests for these comparisons, as appropriate, based on the distributions of the variables.

Next, the simplified version of our previously developed gradient boosted machine model, which was derived only using UC data, was applied to the UC internal validation cohort and the LUMC and NUS external validation cohorts. As previously described, the originally published gradient boosted machine model was developed using discrete time survival analysis, included 97 variables, and was developed and validated solely using UC data.^10^ This model was simplified to 59 variables, with model development performed as described in the prior publication^10^ using the same derivation cohort, with 10-fold cross-validation in the derivation data used to tune the model hyperparameters. Predictors in the simplified model include demographic characteristics, vital signs, routine chemistry and hematology laboratory values, trends of vital sign and laboratory values (eg, highest heart rate in previous 24 hours), and nursing documentation (eg, Braden score) (eTable 1 in the Supplement). Missing data were handled as previously described, with the median (for continuous data) or mode (for categorical data) by location being imputed for missing predictor values that remained after carry-forward imputation.^10^ eFigure 1 in the Supplement illustrates the variable importance plot for the top 15 variables in the simplified model developed from UC data. Of note, this is the same simplified model that is now running prospectively at UC as part of an National Institutes of Health–funded clinical trial.^15^ The simplified model, which was developed only in the previously described UC derivation cohort, was used to produce predicted probabilities for every new observation (eg, new vital sign or laboratory value) in the 3 validation cohorts. These probabilities were used to calculate the area under the receiver operating characteristic curve (AUC) using the trapezoidal method, with the Delong method for confidence intervals.^17^ For accuracy calculations, probabilities were calculated for every observation until the event of interest occurred or the patient was discharged.

The primary outcome of this study was the development of SCr-based stage 2 AKI within 48 hours of each observation. Accuracy metrics at individual probability thresholds were also calculated using the maximum score during the admission prior to the outcome of interest or discharge. Secondary outcomes included the development of stage 1 AKI, stage 3 AKI, receipt of KRT, and inpatient mortality. Subgroup analyses were performed by hospital location (ICU vs ward), admission SCr concentration strata, and time in an operating room. All analyses were performed using Stata version 15.1 (StataCorp) and R version 3.6.1 (The R Project for Statistical Computing). Statistical significance was set at *P* < .05, and all tests were 2-tailed.

## Results

The final cohort included 495 971 adult patient admissions (mean [SD] age, 63 [18] years; 87 689 [17.7%] African American; and 266 866 [53.8%] women) across 6 hospitals at 3 health systems. Exclusions leading to this cohort were previously published for UC^10^ and are found in eFigure 2 in the Supplement for LUMC and NUS. Compared with the other 2 cohorts, patient admissions from UC were more likely to be younger (mean [SD] age: LUMC, 58.6 [17.2] years; NUS, 67.4 [17.7] years; UC, 56.6 [17.8] years; *P* < .001) and African American patients (LUMC, 45 512 [22.7%]; 17 940 [7.3%]; UC, 24 237 [50.0%]; *P* < .001) (Table 1). While statistically significant differences in the admission SCr and blood urea nitrogen (BUN) concentrations were seen across cohorts, the numerical differences were small. The UC internal validation cohort included 48 463 patient admissions, 6935 (14.3%) of whom developed at least stage 1 AKI, 1664 (3.4%) who developed stage 2 or 3 AKI, and 332 (0.7%) requiring KRT. Among the 200 613 patients included in the LUMC cohort, 27 352 (13.6%) developed at least stage 1 AKI, 5722 (2.8%) developed stage 2 or 3 AKI, and 672 (0.3%) required KRT. Among the 246 895 patients in the NUS cohort, 20 473 (8.3%) developed any AKI, 3499 (1.4%) developed stage 2 or 3 AKI, and 440 (0.2%) received KRT. eFigure 3 in the Supplement provides the cumulative incidence plots for all individual stages of AKI and receipt of KRT over time across all 3 cohorts.

**Table 1.** Clinical Demographics and Outcomes of the 3 Patient Cohorts With 495 971 Total Patients

Characteristic	Cohort, No. (%)			*P* value
	LUMC (n = 200 613)	NUS (n = 246 895)	UC (n = 48 463)	
Age, mean (SD), y	58.6 (17.2)	67.4 (17.7)	56.6 (17.8)	<.001
African American	45 512 (22.7)	17 940 (7.3)	24 237 (50)	<.001
Female	100 987 (50.3)	139 947 (56.7)	25 932 (53.5)	<.001
Admission serum creatinine, mean (SD), mg/dL	1.1 (0.4)	1.0 (0.4)	1.0 (0.5)	<.001
Admission blood urea nitrogen, mean (SD), mg/dL	16.9 (11.8)	19.6 (13)	17.9 (12.1)	<.001
Patients developing any AKI				
Stage 1	27 352 (13.6)	20 473 (8.3)	6935 (14.3)	<.001
Stage 2	5711 (2.8)	3499 (1.4)	1664 (3.4)	
Stage 3	2575 (1.3)	1406 (0.6)	775 (1.6)	
Receipt of dialysis more than 48 h after initial serum creatinine measurement	672 (0.3)	440 (0.2)	332 (0.7)	<.001
Length of hospital stay, median (IQR), d	2.8 (1.3-5.8)	3 (1.7-4.8)	3.9 (2.1-6.7)	<.001
Location of AKI				
Ward	16 007 (8.0)	14 540 (5.9)	4818 (9.9)	<.001
ICU	10 434 (5.2)	4735 (1.9)	1949 (4.0)	
Emergency department or other	911 (0.5)	1198 (0.5)	168 (0.3)	
ICU admission during stay	49 803 (24.8)	33 869 (13.7)	10 059 (20.8)	<.001
Operating room during stay	61 813 (30.8)	61 940 (25.1)	13 442 (27.7)	<.001
Inpatient mortality	4286 (2.1)	3215 (1.3)	1050 (2.2)	<.001

Abbreviations: AKI, acute kidney injury; ICU, intensive care unit; IQR, interquartile range; LUMC, Loyola University Medical Center; NUS, Northshore University Health System; UC, The University of Chicago.

SI conversion factors: To convert blood urea nitrogen to millimoles per liter, multiply by 0.357; serum creatinine to micromoles per liter, multiply 88.4.

eTable 2 in the Supplement provides the demographic characteristics and outcome data for all 3 cohorts, stratified by those with and without AKI. Compared with patients who did not develop AKI, those who did were more likely to be older (eg, mean [SD] age in NUS cohort, 66.8 [17.9] years vs 73.2 [14.7] years; *P* < .001); male (96 711 of 226 442 [42.7%] vs 10 226 of 20 473 [49.9%]; *P* < .001), and have higher mean (SD) admission SCr (1 [0.4] mg/dL vs 1.2 [0.6] mg/dL; *P* < .001) and BUN values (19 [12.5] mg/dL vs 26.1 [16.8] mg/dL [to convert to millimoles per liter, multiply by 0.357]; *P* < .001). Compared with patients who did not develop AKI, those who did were more likely to have been in an operating room (eg, LUMC cohort: 49 875 of 173 261 [28.8%] vs 11 938 of 27 352 [43.6%]; *P* < .001) or ICU (34 009 [19.6%] vs 15 794 [57.7%]; *P* < .001), had significantly longer median (interquartile range) hospital lengths of stay (2.4 [1.2-4.7] vs 7.9 [4.4-14.9]; *P* < .001), and had higher inpatient mortality (1403 [0.8%] vs 2883 [10.5%]; *P* < .001).

Model discrimination results for the prediction of all stages of AKI and the need for KRT in the next 48 hours across all 3 cohorts are shown in Table 2. The AUCs were the same or slightly higher in the UC cohort for all outcomes. The model predicted the development of stage 2 AKI within 48 hours with an AUC of 0.86 (95% CI, 0.86-0.86) in the UC cohort, 0.86 (95% CI, 0.86-0.86) in the NUS cohort, and 0.85 (95% CI, 0.84-0.85) in the LUMC cohort. The model provided excellent discrimination of those needing KRT within 48 hours, with AUCs of 0.95 or higher in all 3 cohorts. Table 3 provides the AUCs for the prediction of stage 2 AKI in the next 48 hours across all 3 cohorts stratified by patient location, admission SCr concentration, and prior operating room status. In the UC and LUMC cohorts, the model had slightly higher discrimination for the prediction of stage 2 AKI for patients in the ICU compared with ward patients, although these differences were small (difference, 0.01-0.02). The model had very similar discrimination for the prediction of stage 2 AKI in the next 48 hours on the wards in all 3 cohorts. In all 3 cohorts, the model performed better among patients with higher admission SCr concentrations, performing best among those with an admission SCr concentration between 2.0 and 2.9 mg/dL. In all subgroups across all sites, the AUC for the development of stage 2 AKI in the next 48 hours was greater than 0.80. eTable 3 in the Supplement provides the AUCs for the prediction of stage 2 AKI in the next 24 hours across all 3 cohorts in the same subgroups, which were numerically higher than the results for predicting events within 48 hours. For example, for patients in the NUS with admission SCr concentrations of 2.0 to 2.9, the model had an AUC of 0.93 (95% CI, 0.92-0.93). Subgroup analyses looking at postoperative patients demonstrated that the algorithm performed nearly identically among those who did and did not previously go to an operating room (Table 3; eTable 3 in the Supplement). Table 3 also shows that, in all 3 cohorts, the model provided an AUC of greater than 0.84 for the prediction of postoperative stage 2 AKI in the next 48 hours. Calibration plots for all 3 sites are shown in eFigure 4 in the Supplement, which mimics how the model was derived using 12-hour blocked data. The model was well calibrated at all sites, except for the highest risk decile at UC and the top 2 risk deciles at LUMC and NUS.

**Table 2.** AUC for the Development of AKI and the Receipt of KRT Within the Next 48 Hours^a^

Outcome	AUC (95% CI), by cohort		
	LUMC (n = 200 613)	NUS (n = 246 895)	UC (n = 48 463)
AKI in 48 h			
≥Stage 1	0.67 (0.67-0.67)	0.69 (0.69-0.69)	0.72 (0.71-0.72)
≥Stage 2	0.85 (0.84-0.85)	0.86 (0.86-0.86)	0.86 (0.86-0.86)
≥Stage 3	0.91 (0.91-0.91)	0.92 (0.92-0.92)	0.92 (0.92-0.92)
Receipt of KRT			
In 24 h	0.96 (0.96-0.96)	0.96 (0.95-0.96)	0.97 (0.97-0.97)
In 48 h	0.95 (0.95-0.95)	0.95 (0.94-0.95)	0.96 (0.96-0.96)
In 72 h	0.94 (0.94-0.94)	0.93 (0.93-0.93)	0.94 (0.94-0.94)

Abbreviations: AKI, acute kidney injury; AUC, area under the receiver operating characteristic curve; LUMC, Loyola University Medical Center; NUS, Northshore University Health System; KRT, kidney replacement therapy; UC, The University of Chicago.

^a^ A total of 27 532, 20 473, and 6935 admissions developed stage 1 AKI, 5711, 3499, and 1664 developed stage 2 AKI, 2575, 1406, and 775 developed stage 3 AKI, and 672, 440, and 332 received KRT at LUMC, NUS , and UC, respectively.

**Table 3.** AUCs for the Model to Predict Stage 2 AKI in the Next 48 Hours in All Cohorts Stratified by Patient Location, Admission Serum Creatinine Level, and Time in Operating Room

Variable	AUC for predicting stage 2 AKI within 48 h (95%CI), by cohort		
	LUMC (n = 200 613)	NUS (n = 246 895)	UC (n = 48 463)
Patient location			
Ward	0.80 (0.80-0.80)	0.84 (0.84-0.84)	0.83 (0.83-0.83)
Intensive care unit	0.82 (0.82-0.82)	0.83 (0.83-0.83)	0.84 (0.84-0.85)
Admission serum creatinine, mg/dL			
<1.0	0.81 (0.81-0.82)	0.83 (0.83-0.83)	0.84 (0.84-0.84)
1.0 to <2.0	0.86 (0.86-0.86)	0.88 (0.88-0.88)	0.88 (0.88-0.88)
2.0-2.9	0.89 (0.88-0.89)	0.89 (0.88-0.89)	0.89 (0.89-0.90)
Time spent in an operating room			
Prior operating room	0.84 (0.84-0.85)	0.86 (0.86-0.86)	0.86 (0.86-0.86)
No prior operating room	0.85 (0.85-0.85)	0.86 (0.86-0.86)	0.86 (0.86-0.86)

Abbreviations: AKI, acute kidney injury; AUC, area under the receiver operating characteristic curve; LUMC, Loyola University Medical Center; NUS, Northshore University Health System; UC, The University of Chicago.

SI conversion factor: To convert serum creatinine to micromoles per liter, multiply by 88.4.

Table 4 demonstrates the sensitivity, specificity, and positive and negative predictive values (PPV and NPV) for each probability cutoff using the maximum score for each admission to predict stage 2 AKI during the admission. Several probability cutoff values provided high sensitivity and specificity, with a cutoff of at least 0.057 providing a sensitivity of 87.1%, an NPV of 99.5%, and a PPV of 27.0% in the UC cohort. Similar or slightly lower accuracy results were seen in the LUMC and NUS cohorts across different thresholds (Table 4). eTable 4 in the Supplement demonstrates the same performance metrics using every observation in the test data sets for whether the outcome occurred within 48 hours at each individual probability cutoff across all 3 cohorts.

**Table 4.** Accuracy and Timing of Detection of Different Probability Cutoffs for Detecting Stage 2 AKI Using the Maximum Score During the Admission Prior to the Event or Discharge

Probability cutoff	Patients, No.^a^	Time to stage 2, median (IQR), h	Sensitivity, %	Specificity, %	PPV	NPV
**LUMC cohort**						
≥0.010	50 860	64 (25-175)	90.1	76.5	10.1	99.6
≥0.030	30 580	49 (23-138)	81.7	86.7	15.2	99.4
≥0.045	24 083	44 (21-120)	76.4	89.9	18.1	99.2
≥0.057	20 520	39 (19-108)	73.3	91.6	20.4	99.2
≥0.075	16 508	35 (17-97)	68.0	93.5	23.5	99.0
≥0.100	12 539	28 (15-83)	60.8	95.3	27.6	98.8
≥0.125	9533	25 (13-71)	53.1	96.7	31.7	98.6
≥0.150	7288	24 (11-64)	45.5	97.6	35.6	98.4
≥0.175	5406	23 (11-55)	37.3	98.3	39.3	98.2
≥0.200	3945	22 (9-48)	29.3	98.8	42.4	98.0
≥0.250	2001	18 (8-35)	17.1	99.5	48.6	97.6
≥0.400	52	11 (4-27)	0.6	100.0	63.5	97.2
**NUS cohort**						
≥0.010	44 439	53 (24-133)	86.1	83.0	6.7	99.8
≥0.030	24 048	44 (22-106)	75.2	91.2	10.8	99.6
≥0.045	18 165	39 (21-95)	69.3	93.5	13.2	99.5
≥0.057	15 137	34.5 (19-85)	64.4	94.7	14.7	99.5
≥0.075	11 748	30 (18-73)	58.3	96.0	17.1	99.4
≥0.100	8632	26 (16-65)	50.9	97.2	20.4	99.3
≥0.125	6339	25 (14-54)	42.9	98.0	23.4	99.2
≥0.150	4675	24 (13-48)	36.5	98.6	26.9	99.1
≥0.175	3375	23 (11-46)	29.1	99.0	29.8	99.0
≥0.200	2374	21 (10-41)	22.8	99.3	33.2	98.9
≥0.250	1066	18 (8-32)	11.8	99.7	38.4	98.8
≥0.400	11	21 (8-34)	0.1	100.0	18.2	98.6
**UC cohort**						
≥0.010	13 756	57.5 (22-183.5)	99.1	74.1	12.0	100.0
≥0.030	7971	38 (11-130)	94.3	86.3	19.6	99.8
≥0.045	6249	31 (9-107)	90.6	89.9	24.1	99.6
≥0.057	5360	27 (6.5-93)	87.1	91.6	27.0	99.5
≥0.075	4379	24 (4-74)	81.4	93.5	30.9	99.3
≥0.100	3367	22 (2-56)	73.0	95.4	36.0	99.0
≥0.125	2627	18 (0-47)	64.8	96.7	41.0	98.7
≥0.150	2054	16 (0-37)	56.4	97.6	45.6	98.4
≥0.175	1561	14 (0-30)	45.8	98.3	48.8	98.1
≥0.200	1177	12 (0-27)	35.6	98.8	50.3	97.7
≥0.250	605	10 (0-25)	20.9	99.4	57.4	97.3
≥0.400	7	18 (2-23)	0.3	100.0	71.4	96.6

Abbreviations: AKI, acute kidney injury; IQR, interquartile range; LUMC, Loyola University Medical Center; NPV, negative predictive value; NUS, Northshore University Health System; PPV, positive predictive value; UC, The University of Chicago.

^a^ Number of patients denotes the number of encounters to reach the threshold before first meeting stage 2 AKI.

The utility of the model as a decision support tool, with an illustration of the percentage of observations that crossed each alert threshold by the sensitivity of that threshold for predicting the development of stage 2 AKI within 48 hours, is shown in the Figure. As shown, relatively fewer alerts would fire at UC and NUS compared with LUMC if a high (≥60%) sensitivity was desired. In a time-to-event analysis, a cutoff of at least 0.057 predicted the later onset of stage 2 AKI a median (IQR) of 27 (6.5-93) hours before the eventual doubling in SCr concentration in the UC cohort, 34.5 (19-85) hours in the NUS cohort, and 39 (19-108) hours in the LUMC cohort. Table 4 provides time-to-event analysis for all cutoffs across all cohorts.

**Figure.**
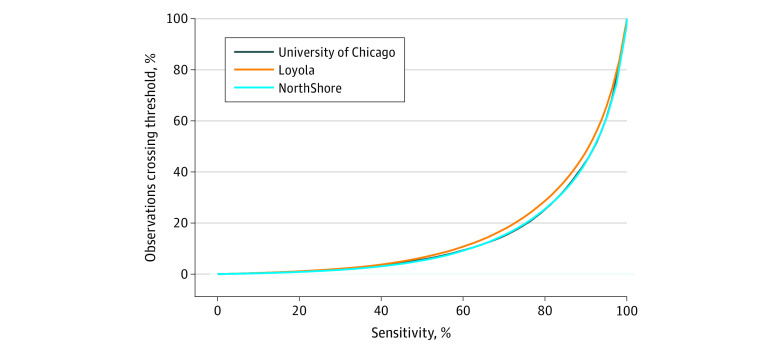
Efficiency Curves for Predicting Stage 2 AKI for All Included Cohorts

## Discussion

In this large, multicenter study across 6 hospitals, 3 health systems, and nearly 500 000 patient admissions, we performed an internal and external validation of a machine learning risk algorithm that predicts the development of AKI across all hospitalized patients. Our findings demonstrate consistent, high discrimination across all sites, hospital locations, and baseline SCr values as well as higher discrimination for the more severe forms of AKI (ie, stage 3 AKI and the need for KRT). Importantly, the model identified patients at risk of AKI nearly a day and a half earlier than the current criterion standard, ie, SCr concentration. This advanced notice could potentially allow for preemptive interventions for patients at high risk of AKI, which could improve outcomes. Our model, which has now been validated in 2 unique, external health systems, uses clinical data that is readily available in the electronic health record and can be implemented for real-time use.^15^

Although model accuracy often decreases during external validation, we found similar results for predicting severe AKI in the internal and external validation cohorts. This may be because AKI is defined using SCr concentrations, and our model included mostly generalizable physiologic variables. As expected, discrimination was slightly higher in the UC internal validation cohort in some subgroups, with the largest difference in performance seen in those with stage 1 AKI. Because this stage of AKI can be affected by benign fluid shifts and fluid administration practices and may not represent true kidney tubular injury, this is likely a less important outcome to predict than more severe stages of AKI.^18,19,20^ Our model’s strengths include its ability to detect AKI in those with and without an elevated SCr concentration at admission. The model demonstrated AUCs greater than 0.90 in the validation cohorts for detecting stage 2 AKI in the next 24 hours in those with an admission SCr concentration greater than 1.0 mg/dL. This discrimination was slightly lower (≥0.85 AUC) for predicting stage 2 AKI in the next 48 hours, regardless of admission SCr concentration.

Other groups have developed electronic risk scores for the detection of AKI. For example, Wilson and colleagues^8^ developed a parsimonious model using retrospective data from 169 859 hospitalized adults across 3 hospitals in the same health care system. They demonstrated that a simple model based on available laboratory data can accurately predict stage 1 AKI with an AUC of 0.74. Of note, this model was built and trained to predict the more common stage 1 AKI rather than the more severe stage 2, which we used as the primary outcome of our model. In addition, Lei and colleagues^14^ used a gradient boosted machine learning model to identify patients at risk of the development of postoperative AKI in a multicenter study. They demonstrated that through the addition of prehospitalization, preoperative, and intra-operative variables, they improved their ability to detect postoperative KDIGO SCr-based stage 1 AKI, with an AUC of 0.82 in their final model.^14^

Recently, Tomasev and colleagues^13^ published a risk score using data from more than 703 000 adult patients in the United States Department of Veterans Affairs Healthcare System. Using a recurrent neural network, they developed an accurate model that could detect KDIGO-defined AKI. Their final model provided a sensitivity of 55.8% and specificity of 82.7%, based on a 2:1 false-to-true alert ratio. However, they used a randomly selected group of patients to serve as their test set, which has been shown to provide optimistic estimates of accuracy compared with external validation.^21^ Additionally, they used deep learning and included 620 000 features, which would be much less interpretable than our model, which had 59 features. Furthermore, the Veteran Affairs data set remains limited because it included only 6.4% female patients and has unknown validity in more diverse settings. In contrast, our cohort was nearly 50% women and included 17.7% African American patients. Furthermore, we included urban and suburban academic centers as well as community nonteaching hospitals, which increases the generalizability of our findings.

Once electronic scores like ours have been developed, their clinical utility needs to be investigated. Several recent studies have demonstrated that early nephrology care in the setting of increased AKI risk or early AKI is associated with improved patient outcomes. Selby and colleagues^22^ performed a multicenter, pragmatic, step-wedge cluster randomized trial across 5 hospitals in the United Kingdom involving the implementation of early AKI e-alerts as well as AKI care bundles and a kidney care–focused educational program for all hospitalized patients. They demonstrated improved quality of kidney care, with shorter length of stay, improved AKI recognition, medication optimization, and fluid assessment.^22^ Similarly, in 2 separate postoperative cohorts, the use of a KDIGO care bundle in patients identified as high risk of severe AKI (via elevation of their urinary tissue inhibitor metalloprotease-2 and insulin-like growth factor binding protein 2 concentration) led to improved patient outcomes.^23,24^ Meersch and colleagues^23^ demonstrated a decrease in the incidence and severity of AKI with the use of a care bundle following cardiac surgery, while Göcze and colleagues^24^ demonstrated decreased AKI severity and reduction in ICU length of stay after major abdominal surgery.^23,24^ As such, it is reasonable to expect that the implementation of an early electronic AKI risk score may similarly improve AKI outcomes. However, these novel tools should be implemented and then thoroughly investigated to determine their utility.

Using a tool like ours would involve implementing an intervention the first time a patient reaches a unique risk threshold to augment our ability to prevent AKI. Table 4 demonstrates the test performance for the first time patients meet unique probability cutoffs. As shown with these results, there are several thresholds with adequate PPV and sensitivity values that could be used in clinical practice. Future work to determine the optimal threshold for clinical action that balances detection rates and false alarms, which will require interventional trials, is needed.

### Limitations

Our study has limitations. We only defined AKI through changes in SCr concentration because of the inability to obtain accurate hourly urine output measurements in all hospitalized patients to comply with KDIGO definitions.^4^ However, this is in line with several other previously published AKI risk scores.^8,13^ Additionally, given the limitations of all 3 data sets (eg, only having access to inpatient data), we defined baseline SCr concentration using the admission values as opposed to outpatient values. This is similar to how we developed our original models and is in line with current standards in AKI research.^9,10,25^ Future study is needed to determine how this assumption affects model accuracy. We excluded patients without any SCr measured during their admission, because whether they developed AKI was unknowable, so our model does not apply to these patients. Another limitation is that our model overpredicted risk for the highest decile of patients, as shown in the calibration plot (eFigure 4 in the Supplement). However, in clinical practice, our focus is in identifying the highest risk patients, so the ordering of patients (as it relates to discrimination) is more important to our clinical workflow than calibration. Finally, the external validation cohorts were mostly teaching hospitals, and all were in Illinois. However, the comparable results, diverse patient populations, and reliance on mostly physiological variables (as opposed to variables such as billing codes, which can vary across hospitals and over time), suggest that our model is likely to be generalizable to a number of other settings.

## Conclusions

In this study, we internally and externally validated a novel machine learning risk score for the prediction of AKI across all hospital settings. This tool, which includes patient demographic characteristics, vital signs, laboratory values, and nursing assessments, can be used to identify patients at increased risk of the development of severe AKI and the need for KRT. Pairing this risk score with early, kidney-focused care may improve outcomes in the patients at the highest risk of the development of AKI.

## Notes

Supplement. **eFigure 1. **Variable Importance Plot for the Simplified Model Developed in the University of Chicago Derivation Cohort **eFigure 2. **Consort Diagram for the 2 External Validation Cohorts **eFigure 3. **Cumulative Incidence Plots for Stages of AKI and Receipt of Dialysis Across All 3 Study Cohorts **eFigure 4. **Calibration Plots for Stage 2 AKI Across All 3 Study Cohorts **eTable 1.** Variables Included in the Gradient Boosted Machine Prediction Model **eTable 2.** Characteristics and Outcome Comparisons Between Those With and Without AKI Across Sites **eTable 3. **Area Under the Receiver Operating Characteristic Curve for the Model to Predict Stage 2 AKI in the Next 24 Hours in All Cohorts, Stratified by Patient Location, Admission Serum Creatinine Level, and Time in Operating Room **eTable 4. **Accuracy and Timing of Detection of Different Probability Cutoffs for Detecting Stage 2 AKI Within the Next 48 Hours Using All Calculated Risk Scores During the Admission Prior to the Event or Discharge
