# Supplementary material for: Internal and External Validation of a Machine Learning Risk Score for Acute Kidney Injury
Source: JAMA Netw Open. 2020 Aug 11;3(8):e2012892. doi: 10.1001/jamanetworkopen.2020.12892 (PMC7420241; doi:10.1001/jamanetworkopen.2020.12892)
Supplement: Supplement. — eFigure 1. Variable Importance Plot for the Simplified Model Developed in the University of Chicago Derivation Cohort eFigure 2. Consort Diagram for the 2 External Validation Cohorts eFigure 3. Cumulative Incidence Plots for Stages of AKI and Receipt of Dialysis Across All 3 Study Cohorts eFigure 4. Calibration Plots for Stage 2 AKI Across All 3 Study Cohorts eTable 1. Variables Included in the Gradient Boosted Machine Prediction Model eTable 2. Characteristics and Outcome Comparisons Between Those With and Without AKI Across Sites eTable 3. Area Under the Receiver Operating Characteristic Curve for the Model to Predict Stage 2 AKI in the Next 24 Hours in All Cohorts, Stratified by Patient Location, Admission Serum Creatinine Level, and Time in Operating Room eTable 4. Accuracy and Timing of Detection of Different Probability Cutoffs for Detecting Stage 2 AKI Within the Next 48 Hours Using All Calculated Risk Scores During the Admission Prior to the Event or Discharge [file jamanetwopen-3-e2012892-s001.pdf]

Supplementary Online Content

Churpek MM, Carey KA, Edelson DP, et al. Internal and external validation of a machine learning risk score for acute kidney injury. *JAMA Netw Open*. 2020;3(8):e2012892. doi:10.1001/jamanetworkopen.2020.12892

eFigure 1. Variable Importance Plot for the Simplified Model Developed in the University of Chicago Derivation Cohort
eFigure 2. Consort Diagram for the 2 External Validation Cohorts
eFigure 3. Cumulative Incidence Plots for Stages of AKI and Receipt of Dialysis Across All 3 Study Cohorts
eFigure 4. Calibration Plots for Stage 2 AKI Across All 3 Study Cohorts
eTable 1. Variables Included in the Gradient Boosted Machine Prediction Model
eTable 2. Characteristics and Outcome Comparisons Between Those With and Without AKI Across Sites
eTable 3. Area Under the Receiver Operating Characteristic Curve for the Model to Predict Stage 2 AKI in the Next 24 Hours in All Cohorts, Stratified by Patient Location, Admission Serum Creatinine Level, and Time in Operating Room
eTable 4. Accuracy and Timing of Detection of Different Probability Cutoffs for Detecting Stage 2 AKI Within the Next 48 Hours Using All Calculated Risk Scores During the Admission Prior to the Event or Discharge

This supplementary material has been provided by the authors to give readers additional information about their work.

© 2020 Churpek MM et al. *JAMA Network Open.*

ONLINE SUPPLEMENT

eFigure 1. Variable Importance Plot for the Simplified Model Developed in the University of Chicago Derivation Cohort

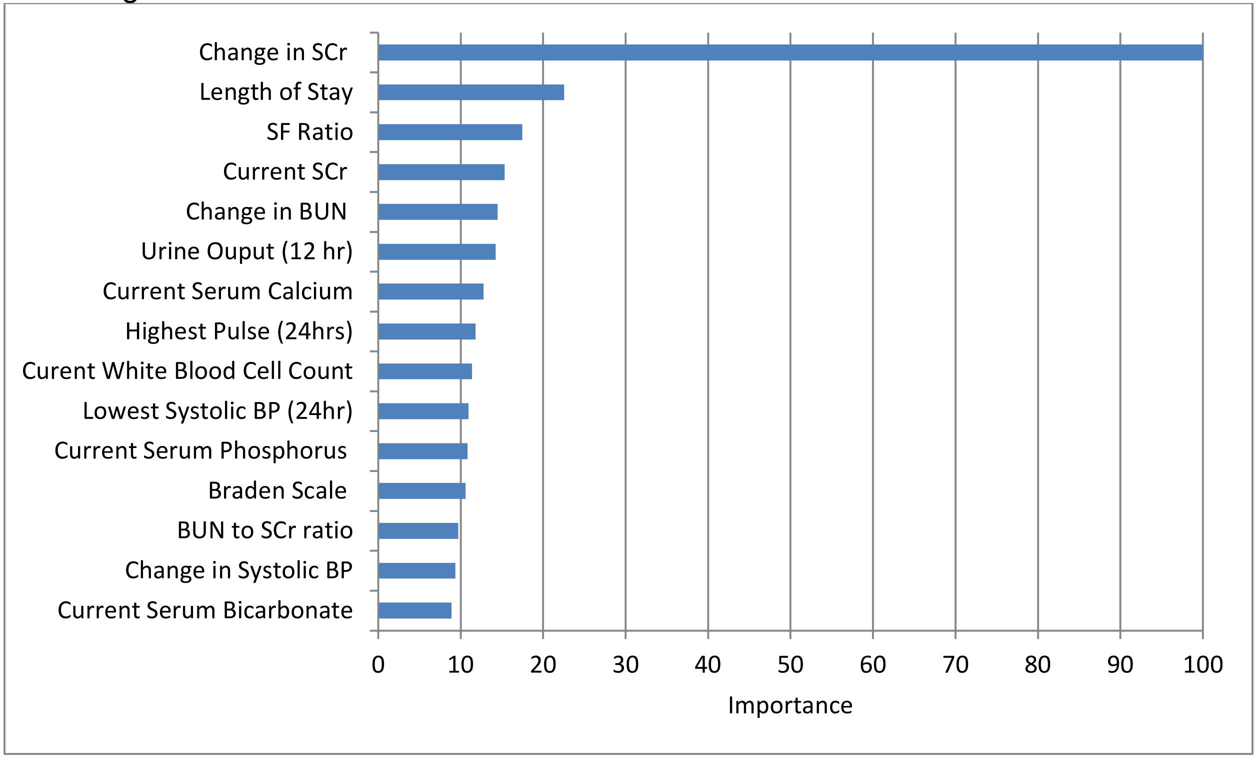

SCr- Serum Creatinine, SF ratio- ratio of oxygen saturation in arterial blood to the percentage of oxygen in inspired air (S_aO_2:FiO_2), BUN- Blood Urea Nitrogen, BP- Blood Pressure, Braden Scale - predicts the risk for developing a hospital or facility acquired pressure ulcer/injury

© 2020 Churpek MM et al. *JAMA Network Open.*

eFigure 2. Consort Diagram for the 2 External Validation Cohorts
This figure demonstrates the size of the LUMC and NUS cohorts and rationale for those who were excluded as well as the acute kidney injury outcomes for those in the final cohort

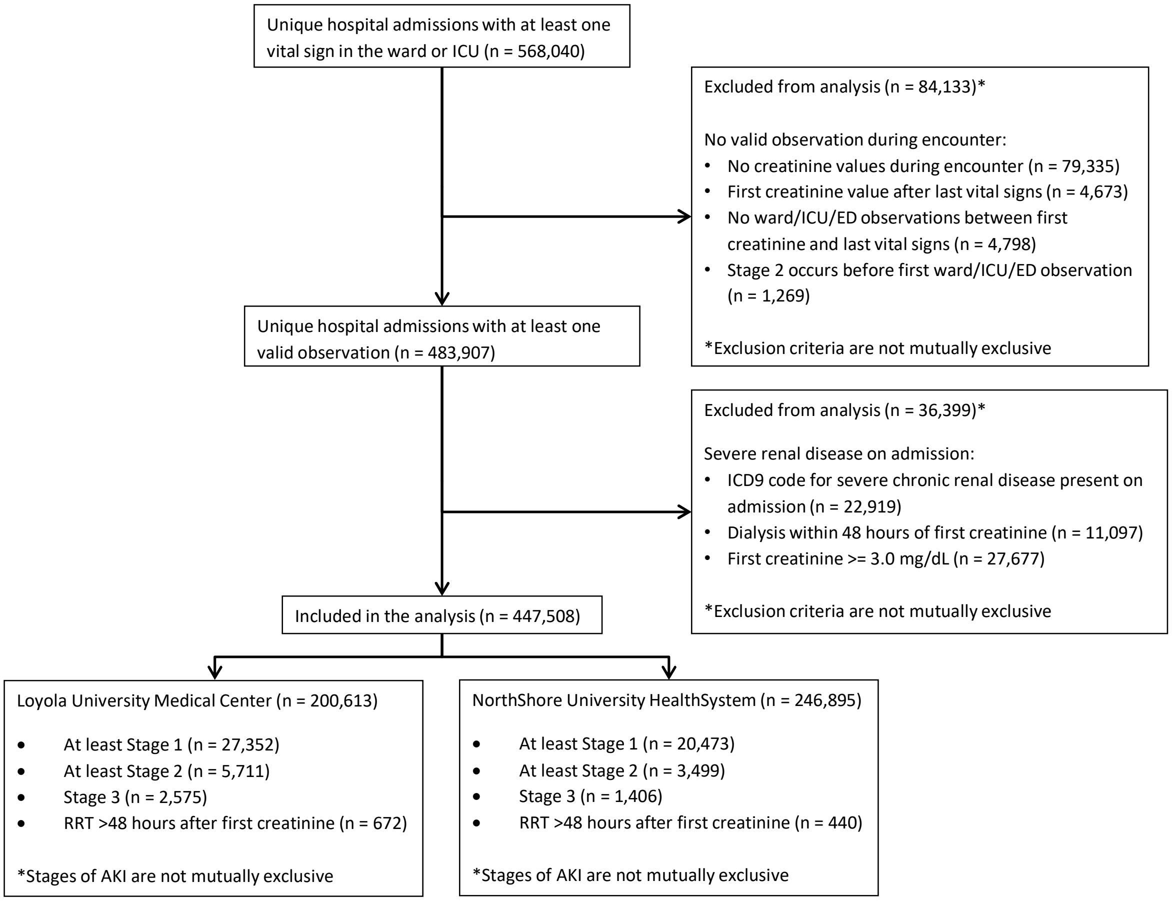

© 2020 Churpek MM et al. *JAMA Network Open.*

eFigure 3. Cumulative Incidence Plots for Stages of AKI and Receipt of Dialysis Across All 3 Study Cohorts

Each plot demonstrates the cumulative incidence of a specific AKI Stage (1,2 or 3) or receipt of dialysis over the first 10 days of an individual's admission. We plotted the incidence of outcome over time, with the denominator being the total number of patients who eventually go on to develop the specific outcome.

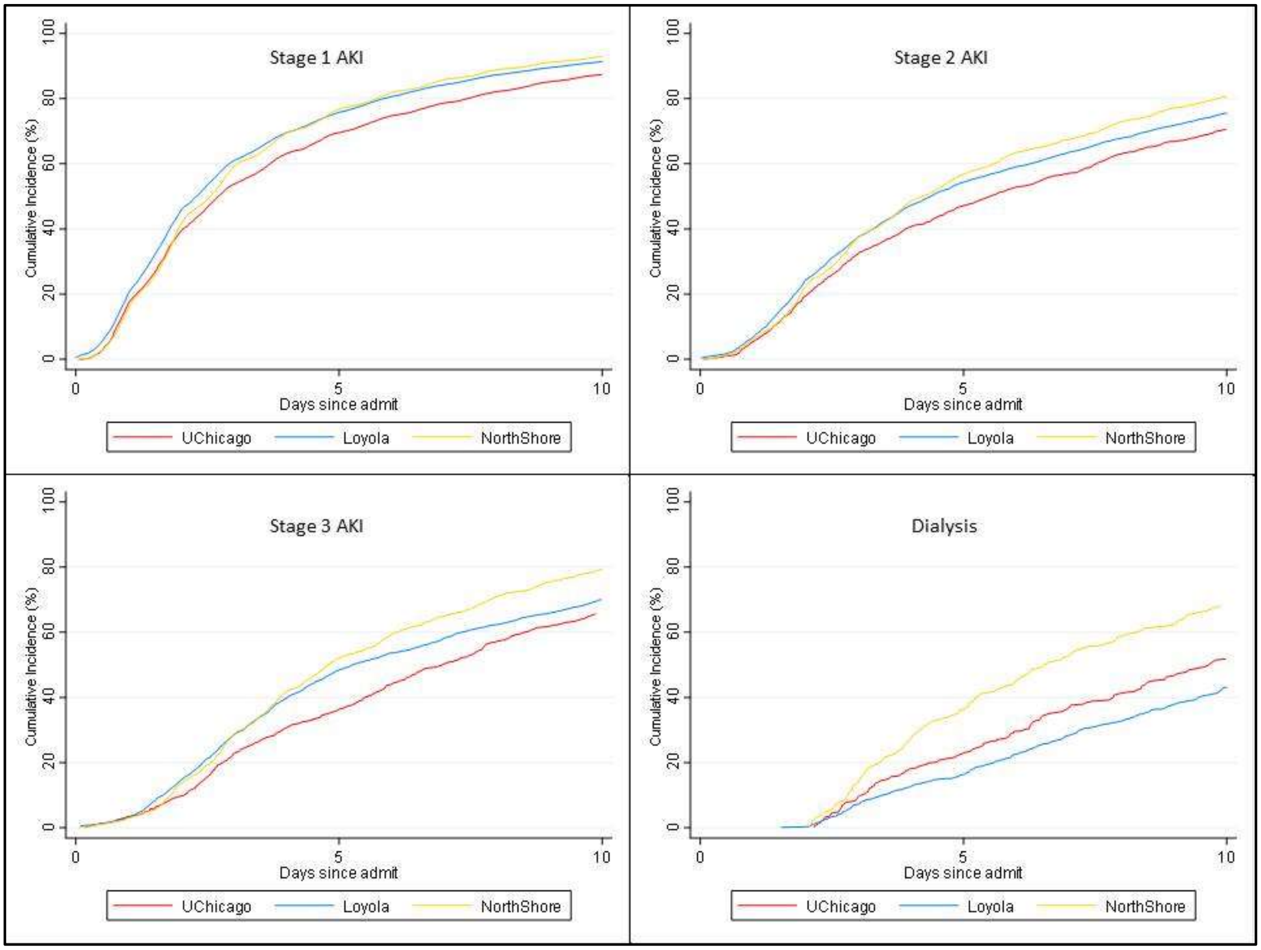

© 2020 Churpek MM et al. *JAMA Network Open.*

eFigure 4. Calibration Plots for Stage 2 AKI Across All 3 Study Cohorts

<table>
<tr><th>Records</th><th>Stage 2 AKI in 12 hours</th></tr>
<tr><td>UChicago</td><td>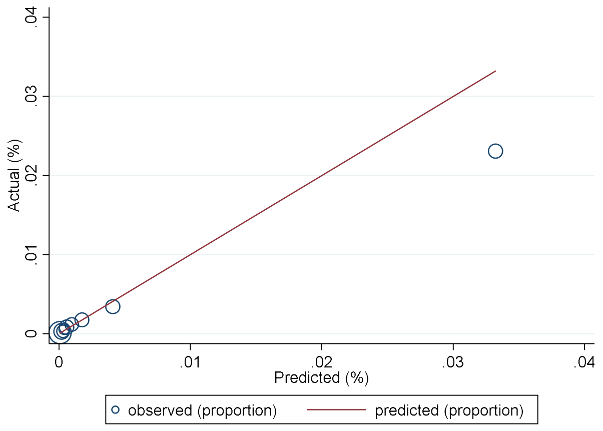
</td></tr>
<tr><td>NorthShore</td><td>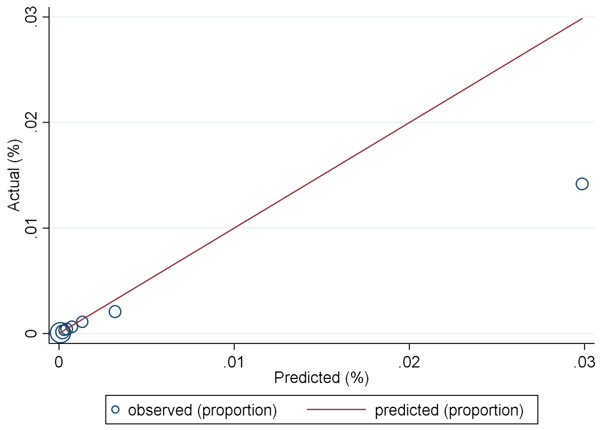
</td></tr>
<tr><td>Loyola</td><td>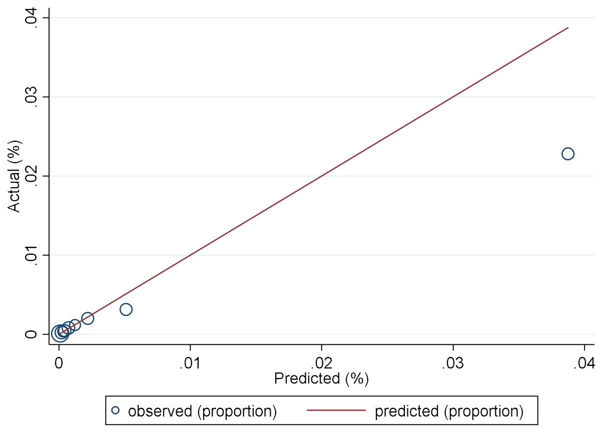
</td></tr>
</table>

© 2020 Churpek MM et al. *JAMA Network Open.*

eTable 1. Variables Included in the Gradient Boosted Machine Prediction Model

Variables included in the model
• Patient Age<br>• Patient Gender<br>• Patient Race<br>• Patient Current Location<br>• Prior ICU Admission During this Admission<br>• Prior Time in the Operating Room During this Admission<br>• Current Length of Stay<br>• Most Recent Vitals (Temperature, Pulse, Respiratory Rate, Blood Pressure, Pulse Pressure Oxygen Saturation)<br>• AVPU (Alert, Voice, Pain, Unresponsive) Score – (Mental Status Measure)<br>• Current Fraction of Inspired Oxygen (FIO2)<br>• SF Ratio - Ratio of Oxygen Saturation in Arterial Blood to the Percentage of Oxygen in Inspired Air (S_aO_2:FiO_2)<br>• Peak and Slope of Pulse in the Last 24 hours<br>• Nadir and Slope of Systolic and Diastolic Pressure in the Last 24 hours<br>• Peak Respiratory Rate in the Last 24 hours<br>• Peak AVPU in the Last 24 hours<br>• Lowest Oxygen Saturation in the Last 24 hours<br>• Clinical Chemistries (Sodium, Potassium , Bicarbonate, Glucose, Calcium, Phosphate, Blood Urea Nitrogen, (BUN) and Serum Creatinine<br>• Anion Gap<br>• BUN: Creatinine Ratio<br>• Blood Counts (White Blood Cell Count, Hemoglobin, Platelets)<br>• Hepatic Function Panel (Total Protein, Albumin, Total Bilirubin, Alkaline Phosphate, Aspartate Aminotransferase (AST))<br>• Serum Lactate<br>• Troponin<br>• Arterial Blood pH<br>• Serum Ketones<br>• Change in BUN, Creatinine, BUN:Creatinine Ratio, Bicarbonate, Sodium , Anion Gap<br>• Braden scale<br>• Total Urine Output in the Last 12 and 24 hours<br>• Indwelling Bladder Catheter (Yes / No)

© 2020 Churpek MM et al. *JAMA Network Open.*

eTable 2. Characteristics and Outcome Comparisons Between Those With and Without AKI Across Sites

	NUS Cohort (n=246,895)			LUMC Cohort (n=200,613)			UofC Cohort (N=48,463)		
Variable	No AKI (n=226,422)	Any AKI (n=20,473)	P-value	No AKI (n=173,261)	Any AKI (N=27,352)	P-value	No AKI (N=41,528)	Any AKI (N=6,935)	P-value
Age, Mean (SD) years	66.8 (17.9)	73.2 (14.7)	<0.001	57.9 (17.4)	62.6 (15.8)	<0.001	55.9 (18)	61.2 (16)	<0.001
Black, n (%)	16466 (7.3%)	1474 (7.2%)	0.70	39818 (23%)	5694 (20.8%)	<0.001	20709 (49.9%)	3528 (50.9%)	0.12
Female, n (%)	129700 (57.3%)	10247 (50.1%)	<0.001	88926 (51.3%)	12061 (44.1%)	<0.001	22529 (54.3%)	3403 (49.1%)	<0.001
Admission serum creatinine (mg/dL), mean (SD)	1 (.4)	1.2 (.6)	<0.001	1 (.4)	1.2 (.5)	<0.001	1 (.4)	1.2 (.6)	<0.001
Admission Blood urea nitrogen (mg/dL)	19 (12.5)	26.1 (16.8)	<0.001	16.1 (10.9)	21.8 (15.4)	<0.001	17 (11.1)	23.6 (15.9)	<0.001
Receipt of Dialysis more than 48 hours after their initial serum creatinine (n, %)	0 (0%)	440 (2.1%)	<0.001	0 (0%)	672 (2.5%)	<0.001	n/a	332 (4.8%)	n/a
Length of Hospital Stay (Days), median (IQR)†	2.8 (1.6, 4.2)	7.1 (4.1, 12)	<0.001	2.4 (1.2, 4.7)	7.9 (4.4, 14.9)	<0.001	4 (2, 6)	8.9 (5, 16.7)	<0.001
Location of AKI, n(%)	n/a		n/a	n/a		n/a	n/a		n/a
Ward		14540 (71%)			16007 (58.5%)			4818 (69.5%)	
ICU		4735 (23.1%)			10434 (38.1%)			1949 (28.1%)	
Emergency Dept./ Other		1198 (5.9%)			911 (3.3%)			168 (2.4%)	
ICU admission during stay, n (%)	25553 (11.3%)	8316 (40.6%)	<0.001	34009 (19.6%)	15794 (57.7%)	<0.001	6770 (16.3%)	3289 (47.4%)	<0.001
Operating Room during stay, n (%)	55823 (24.7%)	6117 (29.9%)	<0.001	49875 (28.8%)	11938 (43.6%)	<0.001	11015 (26.5%)	2427 (35%)	<0.001
Inpatient Mortality, n (%)	1433 (.6%)	1782 (8.7%)	<0.001	1403 (.8%)	2883 (10.5%)	<0.001	359 (.9%)	691 (10%)	<0.001

© 2020 Churpek MM et al. *JAMA Network Open.*

eTable 3. Area Under the Receiver Operating Characteristic Curve for the Model to Predict Stage 2 AKI in the Next 24 Hours in All Cohorts, Stratified by Patient Location, Admission Serum Creatinine Level, and Time in Operating Room

	AUC for predicting stage 2 AKI within 48 h (95%CI), by cohort		
	LUMC (n = 200 613)	NUS (n = 246 895)	UC (n = 48 463)
Patient Location			
Ward	0.85 (0.84, 0.85)	0.88 (0.88, 0.88)	0.87 (0.87, 0.88)
ICU	0.87 (0.87, 0.87)	0.87 (0.87, 0.87)	0.88 (0.88, 0.88)
Admission serum creatinine, mg/dL			
<1.0	0.85 (0.85, 0.85)	0.87 (0.87, 0.87)	0.88 (0.87, 0.88)
1.0 to <2.0	0.91 (0.91, 0.91)	0.92 (0.92, 0.92)	0.92 (0.92, 0.92)
2.0 to 2.9	0.92 (0.92, 0.92)	0.93 (0.92, 0.93)	0.92 (0.92, 0.93)
Time spent in an operating room			
Prior operating room	0.88 (0.88, 0.88)	0.89 (0.89, 0.89)	0.89 (0.89, 0.90)
No prior operating room	0.89 (0.89, 0.89)	0.90 (0.90, 0.90)	0.90 (0.90, 0.90)

© 2020 Churpek MM et al. *JAMA Network Open.*

eTable 4. Accuracy and Timing of Detection of Different Probability Cutoffs for Detecting Stage 2 AKI Within the Next 48 Hours Using All Calculated Risk Scores During the Admission Prior to the Event or Discharge

Probability cutoff	Patients, No.*	Hours to stage 2, median (IQR), h	Sensitivity, %	Specificity, %	PPV	NPV
LUMC cohort						
≥0.010	50860	64 (25-175)	63.3	88.3	9.0	99.2
≥0.030	30580	49 (23-138)	47.3	95.2	15.1	99.0
≥0.045	24083	44 (21-120)	40.8	96.7	18.6	98.9
≥0.057	20520	39 (19-108)	36.9	97.5	21.2	98.8
≥0.075	16508	35 (17-97)	31.5	98.2	24.5	98.7
≥0.100	12539	28 (15-83)	25.1	98.9	28.6	98.6
≥0.125	9533	25 (13-71)	19.8	99.2	32.5	98.5
≥0.150	7288	24 (11-64)	15.4	99.5	36.4	98.5
≥0.175	5406	23 (11-55)	11.7	99.7	40.4	98.4
≥0.200	3945	22 (9-48)	8.7	99.8	44.6	98.4
≥0.250	2001	18 (8-35)	4.3	99.9	51.4	98.3
≥0.400	52	11 (4-27)	0.1	100.0	68.4	98.2
NUS cohort						
≥0.010	44439	53 (24-133)	62.0	90.4	6.3	99.6
≥0.030	24048	44 (22-106)	46.8	96.0	10.8	99.4
≥0.045	18165	39 (21-95)	40.2	97.3	13.5	99.4
≥0.057	15137	34.5 (19-85)	36.0	97.9	15.5	99.3
≥0.075	11748	30 (18-73)	30.5	98.6	18.2	99.3
≥0.100	8632	26 (16-65)	24.3	99.1	21.7	99.2
≥0.125	6339	25 (14-54)	19.0	99.4	24.9	99.2
≥0.150	4675	24 (13-48)	14.6	99.6	27.9	99.1
≥0.175	3375	23 (11-46)	10.7	99.8	31.1	99.1
≥0.200	2374	21 (10-41)	7.5	99.8	34.0	99.0
≥0.250	1066	18 (8-32)	3.0	99.9	37.5	99.0
≥0.400	11	21 (8-34)	0.0	100.0	15.7	99.0
UC						
≥0.010	13756	57.5 (22-183.5)	64.0	89.7	10.3	99.3
≥0.030	7971	38 (11-130)	47.6	95.8	17.2	99.0
≥0.045	6249	31 (9-107)	40.5	97.2	21.0	98.9
≥0.057	5360	27 (6.5-93)	36.4	97.9	23.9	98.8
≥0.075	4379	24 (4-74)	31.0	98.5	27.7	98.7
≥0.100	3367	22 (2-56)	24.8	99.0	32.5	98.6
≥0.125	2627	18 (0-47)	19.2	99.4	36.3	98.5
≥0.150	2054	16 (0-37)	14.6	99.6	39.1	98.4
≥0.175	1561	14 (0-30)	10.6	99.7	42.2	98.4

© 2020 Churpek MM et al. *JAMA Network Open.*

≥0.200	1177	12 (0-27)	7.6	99.8	45.0	98.3
≥0.250	605	10 (0-25)	3.5	99.9	50.3	98.2
≥0.400	7	18 (2-23)	0.0	100.0	66.7	98.2

© 2020 Churpek MM et al. *JAMA Network Open.*
